# Similarities and differences between the ‘cytokine storms’ in acute dengue and COVID-19

**DOI:** 10.1038/s41598-020-76836-2

**Published:** 2020-11-16

**Authors:** Shashika Dayarathna, Chandima Jeewandara, Laksiri Gomes, Gayasha Somathilaka, Deshni Jayathilaka, Vimalahan Vimalachandran, Ananda Wijewickrama, Eranga Narangoda, Damayanthi Idampitiya, Graham S. Ogg, Gathsaurie Neelika Malavige

**Affiliations:** 1 Centre for Dengue Research, Faculty of Medical Sciences, University of Sri Jayawardenapura, Nugegoda, Sri Lanka; 2 National Institute of Infectious Diseases, Angoda, Sri Lanka; 3 Allergy, Immunology and Cell Biology Unit, University of Sri Jayewardenepura, Nugegoda, Sri Lanka; 4 MRC Human Immunology Unit, MRC Weatherall Institute of Molecular Medicine, University of Oxford, Oxford, UK

**Keywords:** Immunology, Microbiology

## Abstract

Severe pneumonia and multiorgan dysfunction in COVID-19 and dengue haemorrhagic fever (DHF) are two diseases that can associate with an altered immune response to the infecting virus. To determine the similarities and differences in the cytokine and chemokine responses in these two infections, we compared responses in patients with varying severity of COVID-19 and acute dengue at different time points of illness. During early disease, patients who proceeded to develop COVID-19 severe pneumonia (SP) and DHF had significantly higher levels of IL-6, IL-10 and MIP3α than those who developed mild illness. The lowest levels of IFNγ in early illness were seen in those who succumbed to their illness due to COVID-19. Levels of serum IL-10 (*p* = 0.0001), IL-6 (*p* = 0.002), MIP-3α (*p* = 0.02) and CD40-L levels (*p* = 0.002) significantly increased from 5 to 9 day of illness to 10–21 day of illness in patients with moderate-to-severe COVID-19, but not in those with mild illness. In contrast, these cytokine/chemokine levels remained unchanged in those with DHF or dengue fever (DF) during febrile and critical phases. Although IL-10 levels were significantly higher in COVID-19 patients with SP, patients with DHF had 25-fold higher levels, whereas IL-6 levels were 11-fold higher in those with COVID-19 SP. IL-10 and other cytokines were evaluated in a larger cohort of patients during early illness (≤ 4 days) who proceeded to develop DF (n = 71) or DHF (n = 64). Of the cytokines evaluated, IL-10 was significantly higher (*p* < 0.0001) in those who went on to develop DHF compared to DF. Low IFNγ response to the SARS-CoV2 and high levels of immunosuppressive IL-10 in both COVID-19 and dengue during early illness are indicators of an altered antiviral response potentially contributing to disease severity.

## Introduction

COVID-19, caused by the SARS-CoV2 virus, has currently infected over 25 million individuals, resulting in over 850,000 deaths worldwide, within a period of 8 months^1^. COVID19 is characterized by a gradual onset of lower respiratory tract symptoms such as sore throat, cough, fever and tiredness, which is self-limiting in a majority of individuals, but progresses to severe pneumonia and death in a significant proportion of individuals^2^. In one study, mild clinical disease was seen in 81% of symptomatic infected individuals, whereas 19% developed severe disease of whom 5% became critically ill^2^. The case fatality rates are 47% in critically ill patients and acute respiratory distress syndrome (ARDS), acute kidney and myocardial injury are the leading causes of death^3^. ARDS is a prominent feature of those who develop severe pneumonia and is thought to occur due to lung injury caused by the production of high levels of inflammatory cytokines^4^. Several studies have shown that the cytokine storm directly correlated with the extent of lung injury, multiorgan failure and mortality in patients^5,6^. Many different types of inflammatory cytokines such as IL-6, IL-2, IL-7, IL-1β and many others have shown to be associated with clinical disease severity^3,5^.

Acute dengue infection is caused by one of the four dengue viruses (DENVs), is asymptomatic in a majority of infected individuals. However, it may manifest as an undifferentiated viral fever, dengue fever (DF), or may progress into severe illness resulting in dengue haemorrhagic fever (DHF) with or without shock^7^. DF that manifest as an acute febrile illness characterized by fever, myalgia, arthralgia is a self-limiting illness in the majority of infected individuals^7^. However, severe dengue including DHF and organ involvement occurs in up to 10–25% of individuals^8,9^. High levels of inflammatory cytokines resulting in a ‘cytokine storm’ is seen in patients with DHF and is thought to contribute to disease pathogenesis and the vascular leak^10–13^. The initial clinical features are somewhat similar in both dengue and COVID19, along with similar laboratory features such as thrombocytopenia and leucopenia^14^, and the occurrence of a ‘cytokine storm’. Gastrointestinal manifestations such as diarrhoea are common and seen in patients with acute dengue and in COVID-19, and sore throat is a feature in around 38% of patients with acute dengue^15–18^. Therefore, at the initial presentation, especially in countries where dengue is endemic, it can be difficult to clinically differentiate dengue from COVID-19, purely based on clinical and laboratory features. In addition, although COVID-19 is predominantly a lower respiratory tract infection, a recent autopsy case series from China has shown that the virus was detected in lymphoid tissue, liver, heart, kidney and the skin with widespread pathological changes, suggesting that the virus can cause a broad systemic infection. Therefore, apart from the presence of a cytokine storm and similarities in clinical features, both infections appear to be able to cause widespread pathological changes in nearly all organs.

In DHF, the patient usually develops vascular leakage resulting in pleural effusions, ascites and/or shock during day 4–6 of illness^19^. This vascular leakage phase, also known as critical phase, only lasts for 24–48 h and the patient then proceeds to the recovery phase^7,20^. Those with milder forms of dengue (DF), proceed to the recovery phase from the febrile phase without experiencing vascular leakage. If untreated the case fatality rates of DHF is around 20% and is usually due to prolonged shock^21^. On the other hand, severe clinical manifestations due to COVID-19 occurs usually during the second week since onset of symptoms^22,23^. Therefore, the timing of the onset of severe disease manifestations are very different in these two infections.

An aggressive inflammatory response resulting in simultaneous release of high levels of many different types of inflammatory cytokines, which results in disease pathogenesis, is known as a cytokine storm^5^. Many inflammatory cytokines and chemokines such as IL-6, IL-1β, IL-8, CCL8, CXCL9, CXCL16, MCP-1 and IP-10 and immunosuppressive cytokines such as IL-10 are elevated in both infections and associate with clinical disease severity^4,11,24–27^. Although both dengue and COVID-19 are associated with a cytokine storm and multiorgan involvement, the pathogenesis, disease course and recovery are very much different. Therefore, in order to develop a better insight into both infections and to understand the role of these cytokines in disease pathogenesis and the changes along the course of illness, we compared the cytokine and chemokine responses in patients with varying severity of acute dengue and acute COVID-19 illness during the acute/febrile phase and the critical phase of illness. As we were able to identify unique cytokine/chemokine signatures associated with dengue and COVID-19, we further proceeded to evaluate the predictive values of relevant cytokines in early acute dengue, in order to determine subsequent disease severity.

## Results

### Cytokine and chemokine responses in COVID-19 patients

**Figure 1:**
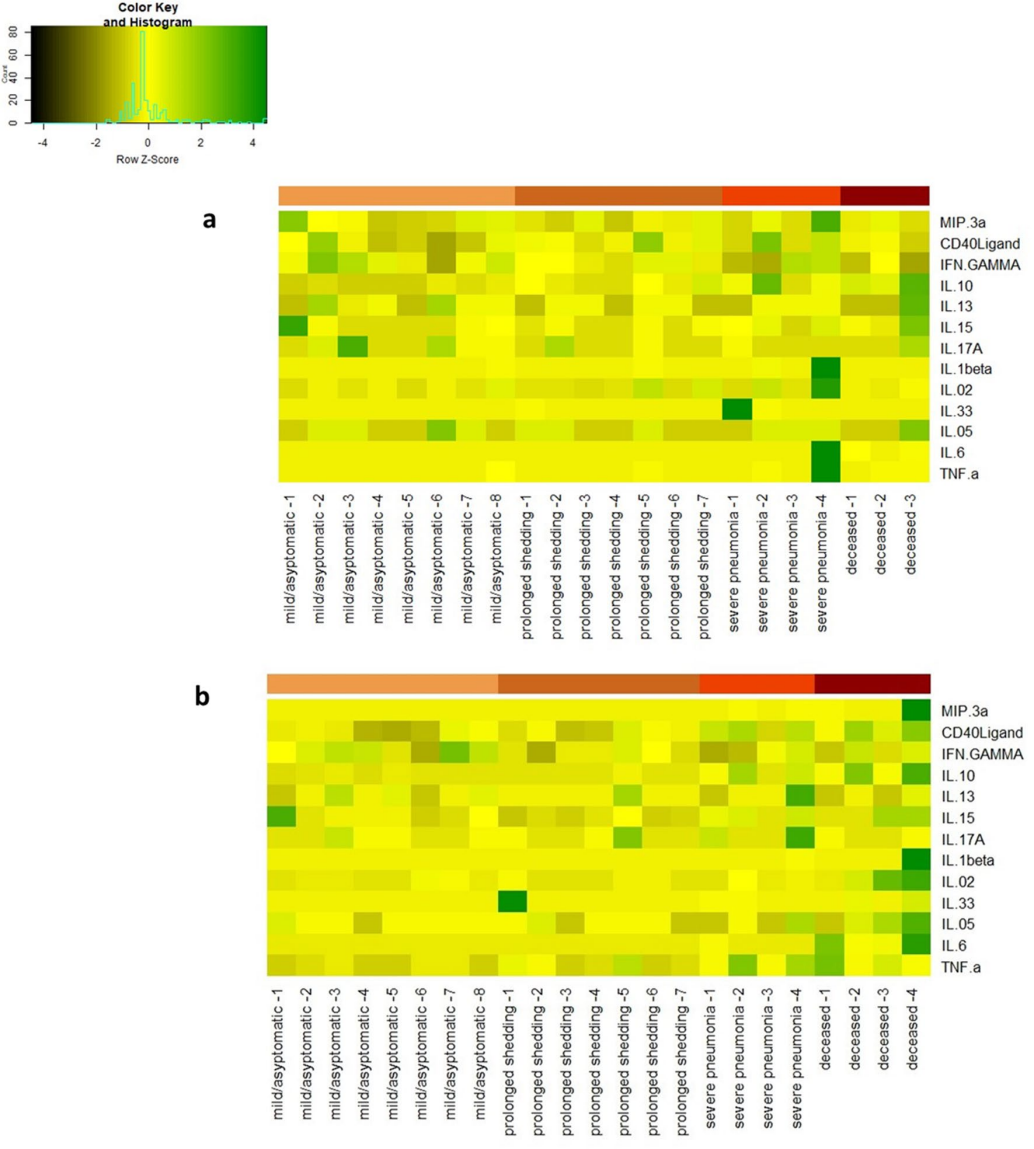
Heat maps representing cytokine and chemokine concentrations in patients with varying severity of COVID19: Each single row represents an individual cytokine or a chemokine level and shown during day 4–9 of illness (time point A) **a**, and during day 10 to 21 of illness (time point B), **b** each column represents a different patient.

Many different cytokines and chemokines were found to be elevated in patients with acute COVID-19 and in dengue (Fig. 1). In patients with COVID-19, the first time point in analysis (A) was between day 4–9 of illness, and the second time point was day 10–21 day of illness (n = 22) (B). As those who had severe disease or prolonged shedding and were hospitalized for over 40 days, a third time point (C) was evaluated between day 35 and 50 days since onset of illness in these individuals (n = 11). The heatmaps of the cytokine levels in those with mild illness (n = 14), severe pneumonia (n = 4) and those who deceased (n = 4) are shown at time point A (Fig. 1A) and time point B (Fig. 1B). The cytokine levels in these different groups at various time points are shown in Supplementary Table 1.

**Figure 2:**
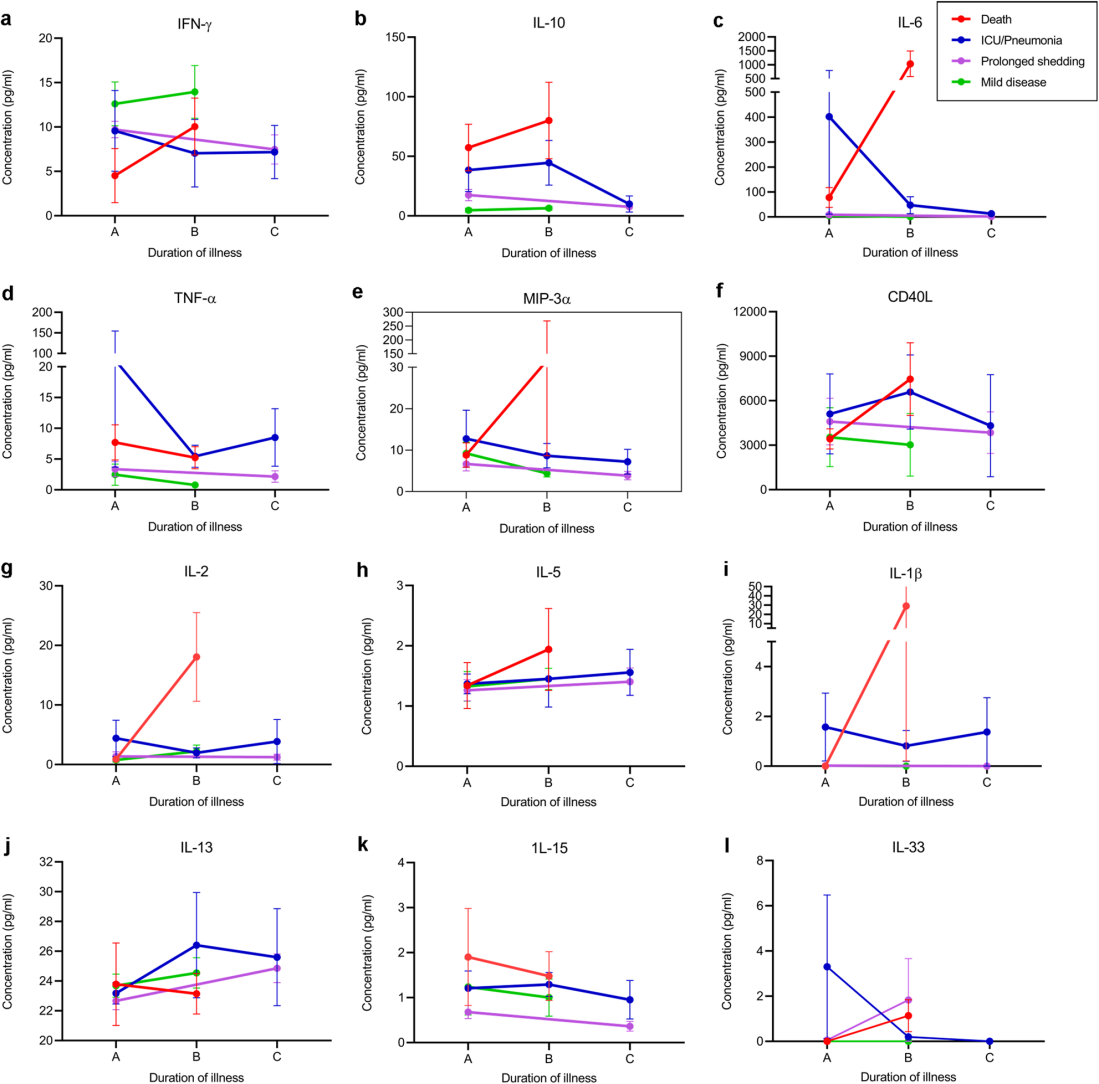
Serum cytokine concentrations in patients with varying severity of COVID-19. Cytokine and chemokine levels were measured by Luminex bead based Th9/Th17/Th 22 discovery performance assay in those who succumbed to their illness (n = 4), those who had severe pneumonia but survived (n = 4), those who had prolonged shedding (n = 7) and those with mild illness (n = 8), during day 4–9 ( time point A, n = 22) and during day 10–21 of illness (time point B, n = 22). Levels were also measured who had prolonged shedding and those with severe pneumonia during day 35–50 illness (time point C, n = 11) (**a**–**l**). The mean cytokine levels with SEM is shown. GM-CSF, IL-4, IL-12p70 and IL-17E were not within detectable range and not included in the figures.

During early illness (1st week of illness), those with mild illness had a tendency to have higher IFNγ levels compared to those who deceased (n = 4), although not significant (*p* = 0.27) (Fig. 2a). The highest IFNγ responses were seen in those with mild infection, which were higher than those who had mild illness, but prolonged shedding. Both Serum IL-10 (*p* = 0.002) and IL-6 levels (*p* = 0.03) were also significantly higher in those who deceased or developed severe pneumonia, than in those with mild illness during early illness (Supplementary Table 1, Fig. 2b,c). IL-10 levels were lowest in those with mild infection, which were significantly lower than in those who had mild illness, but prolonged shedding of the virus (*p* = 0.02).

Serum IL-10 (*p* = 0.0001), IL-6 (*p* = 0.002), MIP-3α (*p* = 0.02) and CD40-L levels (*p* = 0.002) significantly increased from time point A to B (5–9 day of illness to 10–21 day of illness) in deceased individuals and in those who developed severe pneumonia, when compared to those who had mild illness (Fig. 2b–f). No significant changes in TNFα, IL-1β, IL-13 and IL-15 were seen between patients with varying disease severity (Fig. 2d,i–k). Only IL-2 and IL-5 increased in the 4 individuals who deceased at time point B, whereas in all other patients, the levels of these cytokines decreased over the following week (Fig. 2g,h). Detectable levels IL-17E, IL-12p70 and GM-CSF were not seen in patients with COVID-19 at either time point A or B of illness. Very low levels of IL-33 were seen in 2 patients with severe pneumonia, 2 patients who succumbed to their illness and 2 patients with prolonged shedding (Fig. 2l). Low levels of IL-1β were only seen in 2 patients with severe pneumonia and low levels of IL-17A in 2/8 patients with severe pneumonia, and 7/14 in mild illness.

### Comparison of cytokine responses in COVID-19 patients versus patients with DF and DHF

Those who develop severe dengue disease (DHF) due to plasma leakage, enter the critical phase during day 4–7 of illness. The plasma leakage phase lasts for 24–48 h and then patients typically enter the recovery phase^19^. Therefore, by day 10 of illness, even those who develop DHF are in the recovery phase, unless they develop severe organ impairment. As dengue has a more acute course than COVID-19 and as the critical phase occurs between day 5 to 8, for comparison with COVID-19, the acute time point (A) was defined as those with illness of ≤ 4 days duration (day 2 to 4) and the second time point was between day 5 to 7 (B).

**Figure 3:**
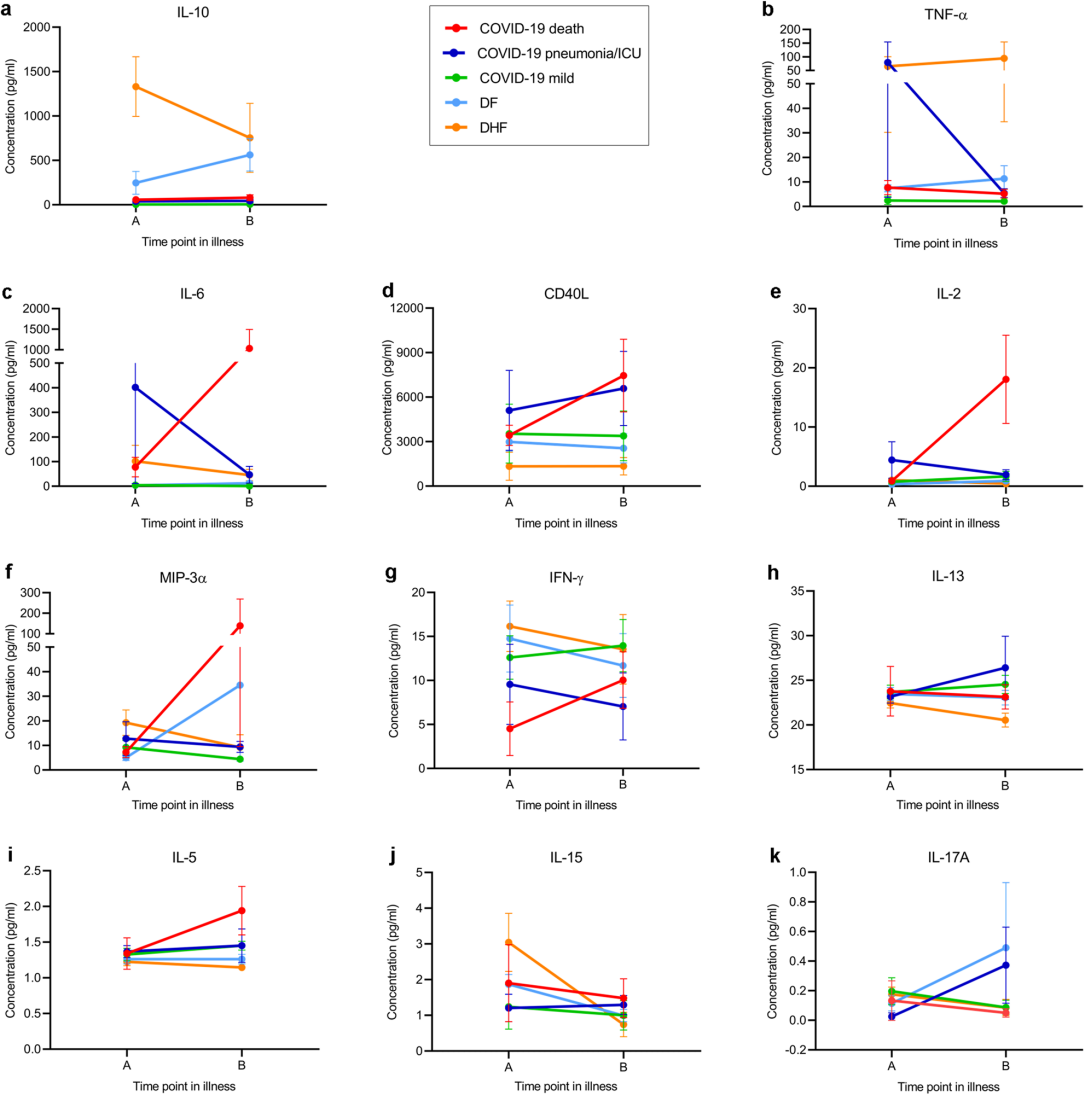
Comparison of serum cytokine concentrations in patients with varying severity of COVID-19 and acute dengue. Cytokine and chemokine levels were measured by Luminex bead based Th9/Th17/Th 22 discovery performance assay in patients with COVID-19 who succumbed to their illness (n = 4), those who had severe pneumonia but survived (n = 4), those with mild illness (n = 8), during day 4 to 9 ( time point A, n = 22) and during day 10–21 of illness (time point B, n = 22) and also in patients with DF (n = 7) and DHF (n = 7) during early illness (time point A, ≤ 4 days of illness) and during the critical phase (time point B, day 5 to 7 of illness). The mean cytokine levels with SEM is shown.

The most striking differences in the cytokines were seen between IL-10, TNFα and IL-6 (Fig. 3). Although patients who developed severe pneumonia in COVID-19 had significantly higher levels of IL-10 than those with milder illness, patients who proceeded to develop DHF, had several fold higher levels during early illness (Fig. 3a). Those who proceeded to develop DHF had significantly higher IL-10 levels (*p* = 0.0003) compared to COVID-19 patients who developed severe illness (Supplementary Table 1 and Fig. 3a). Although TNFα levels were also higher in patients who developed DHF compared to those who developed severe pneumonia, this was not significant (*p* = 0.29) (Fig. 3b). In contrast, serum IL-6 levels and soluble CD40L (sCD40L) levels were higher in patients who developed severe pneumonia compared to those who developed DHF (Supplementary Table 1 and Fig. 3c,d), although not significant. Serum IL-6, sCD40L, IL-5 and IL-2 further rose in time point B in patients who developed severe pneumonia, while no such change was seen in patients with DHF.

### Changes in cytokines and chemokines in patients with DF and DHF

**Figure 4:**
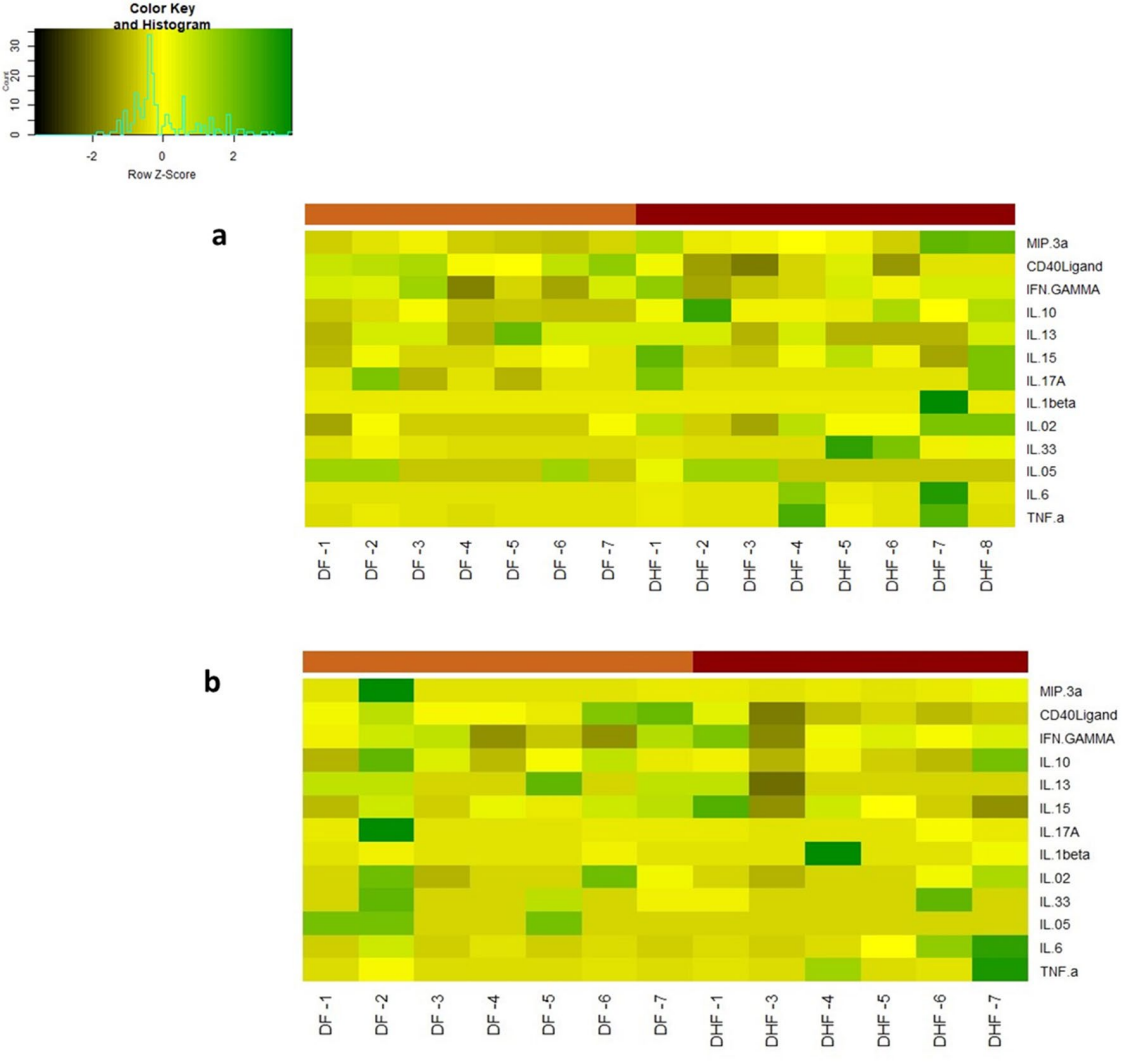
Heat maps representing cytokine and chemokine concentrations in patients with varying severity of acute dengue infection: Each single row represents an individual cytokine or a chemokine level and shown during day ≤ 4 days of illness (time point A) **a**, and during day 5 to 7 of illness (time point B), **b** each column represents a different patient.

We then evaluated the changes in cytokines during early illness (time point A) and during the critical stage (time point B) in patients who proceeded to develop either DF or DHF. The heatmaps of the cytokine levels in those with DF (n = 7), and DHF (n = 7) are shown at time point A (Fig. 4a) and time point B (Fig. 4b).

**Figure 5:**
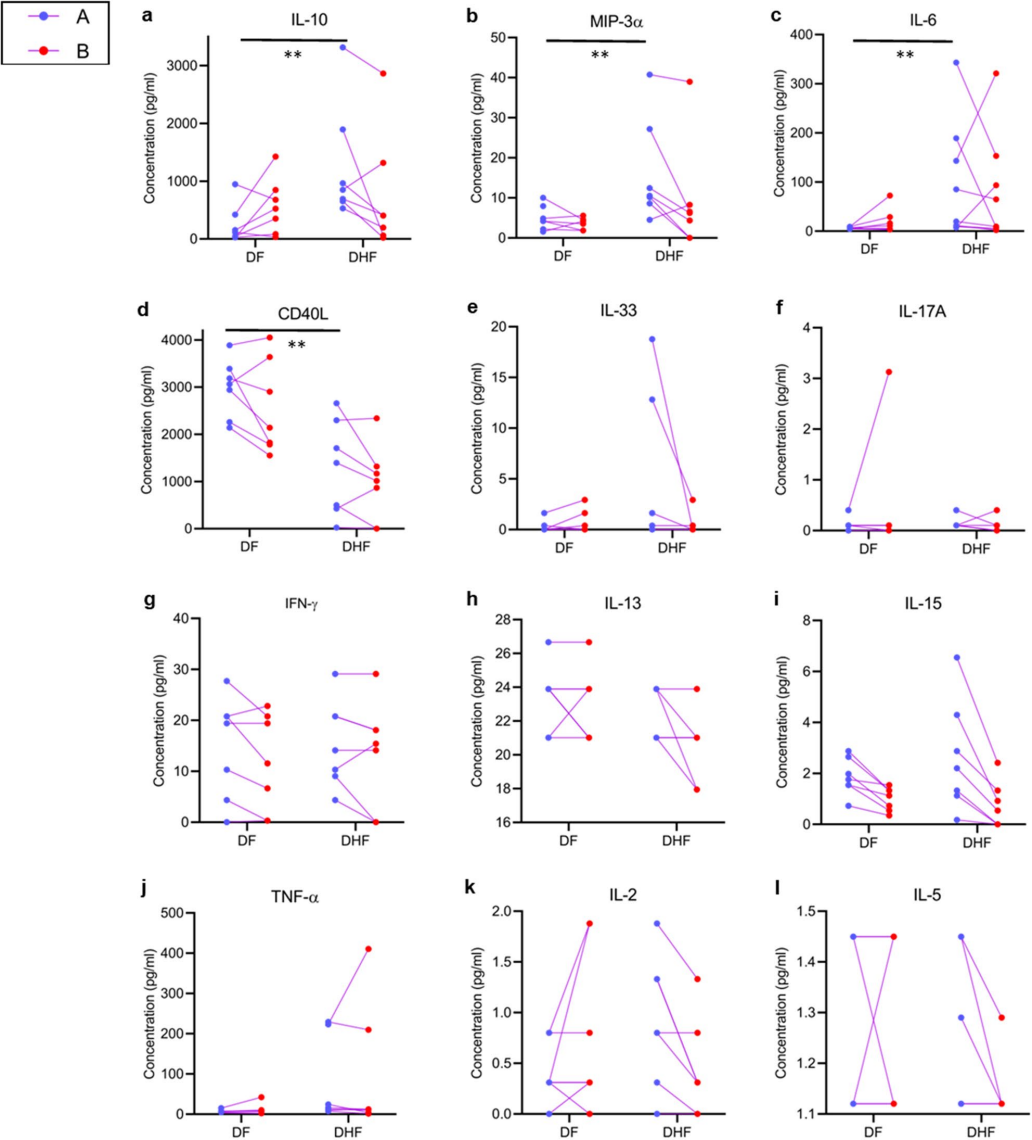
Serum cytokine concentrations in patients with varying severity of acute dengue. Cytokine and chemokine levels were measured by Luminex bead based Th9/Th17/Th 22 discovery performance assay in patients with DF (n = 7) and DHF (n = 7) during early illness (time point A, ≤ 4 days of illness) and during the critical phase (time point B, day 5 to 7 of illness) **a–l**. The mean cytokine levels with SEM is shown. GM-CSF, IL-4, IL-12p70 and IL-17E were not detected and therefore not shown. **p < 0.001.

IL-10 (*p* = 0.007), MIP-3α (*p* = 0.006) and IL-6 (*p* = 0.008) were significantly higher in patients who developed DHF during early illness (Fig. 5a–c) compared to those who developed DF, whereas sCD40L was higher in those with DF (*p* = 0.007) (Fig. 5d). Serum IL-33 was detected in 5/7 patients who developed DHF and 2/7 patients who developed DF during time point A and 2/7 with DHF and 3/7 DF at time point B (Fig. 5e). Very low but detectable IL-17 A levels were produced by all patients with DHF and 5/7 patients with DF during early illness (Fig. 5f). There were no differences in IFNγ, IL-13, IL-15, TNFα, IL-2 and IL-5, although some patients with DHF had higher levels of IL-15 during early illness (Fig. 5g–l). Detectable levels of GM-CSF, IL-12p70 and IL-17E were not seen in these patients at any time point.

### Predictive markers of DHF in early illness

**Figure 6:**
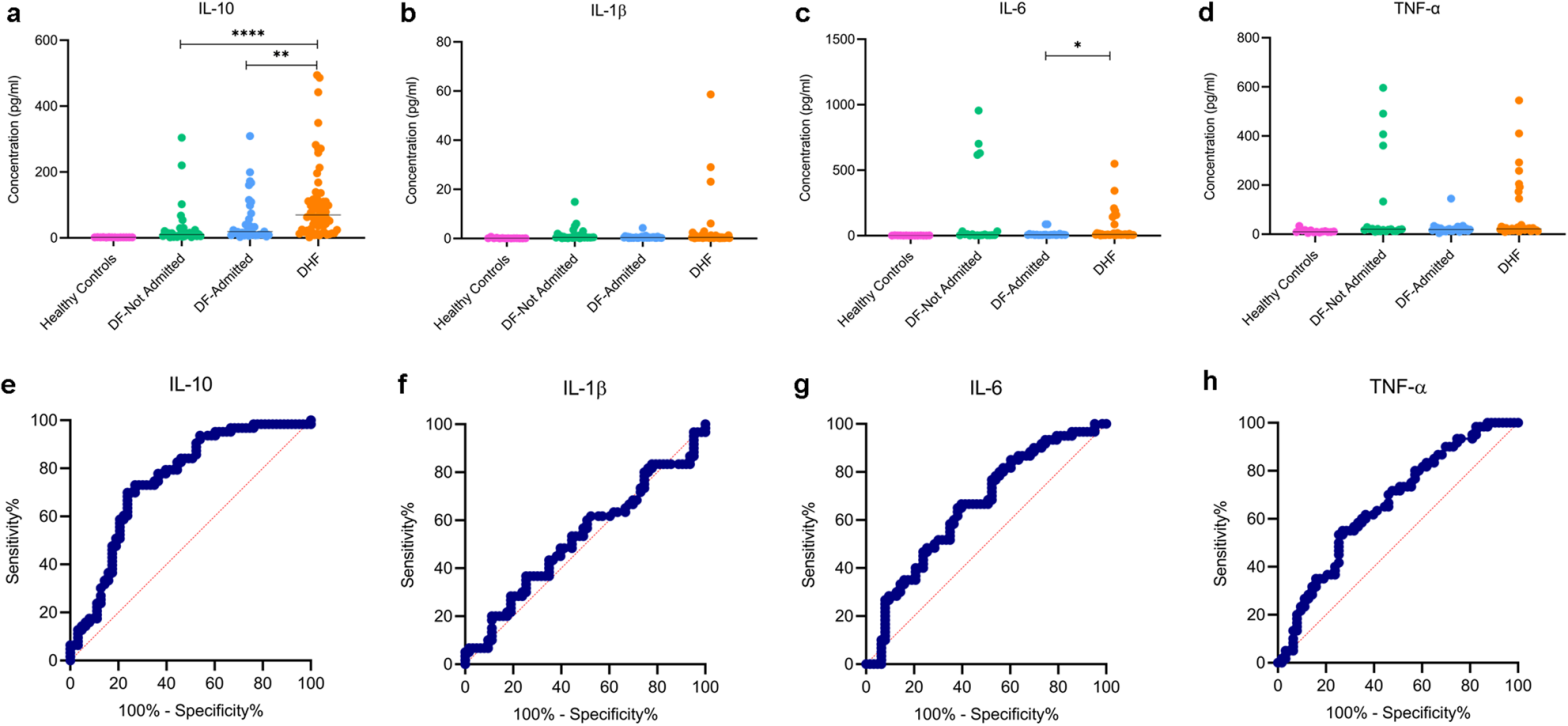
Serum cytokine concentrations in patients with varying severity of acute dengue infection. Serum IL-10 (**a**), IL-1β (**b**), IL-6 (**c**) and TNFα (**d**) was measured by using the ELLA platform in healthy individuals (n = 14), patients with DF who were not admitted to hospital (n = 30), those with DF who were admitted to hospital (n = 36) and in patients who proceeded to develop DHF (n = 64) during ≤ 4 day of illness. ROC curves were generated for each cytokine (**e**–**h**) to calculate the AUC value. *p < 0.05, **p < 0.001, ****p < 0.0001.

As IL-10, IL-6 and TNFα was higher in the febrile phase in those who proceeded to develop DHF and since it has been reported that IL-1β is also associated with increase in vascular permeability^28^, we evaluated the usefulness of these 4 cytokines in a large cohort of patients who proceeded to develop varying degree of clinical disease severity. The levels of these four cytokines were assessed in healthy individuals (n = 14), patients with DF who received ambulatory care as they were not ill enough to receive in-patient care (n = 35), hospitalized patients with DF (n = 36) and those who developed DHF (n = 64). The clinical and laboratory features of these patients are described in Supplementary Table 2. All blood samples were obtained between day 2 and 4 of illness, before any patient developed vascular leak. Serum IL-10 levels were significantly higher (*p* < 0.0001) in those who developed DHF compared to those with DF who were not admitted or admitted (Fig. 6a). There was no significant difference between IL-6, IL-1β and TNFα between the three groups, although those with DHF tend to have higher levels of these cytokines (Fig. 6b–d). 5 patients with DF who were not admitted had high levels of TNFα and IL-6 but not IL-10 (Fig. 6a,c,d). Except for one patient who had diabetes, and another with allergic rhinitis, the other 3 patients did not have any comorbid illnesses and were between the ages of 23–36 years.

As IL-10 was significantly higher in patients who proceeded to develop DHF, we evaluated the usefulness of IL-10 in early illness by assessing the AUC values by comparing levels of those with DHF and DF (both admitted and not admitted grouped together). The AUC was 0.73 (95% CI 0.65–0.82), which was significant (*p* < 0.0001) (Fig. 6e). At IL-10 levels of > 34.3 pg/ml, the sensitivity and specificity of developing DHF was 71.9% and 70% respectively, with a likelihood ratio of 2.4. The AUC values for the other cytokines were not significantly linked to severe disease and were 0.52 for IL-1β (95% CI 0.41–0.52), 0.64 for IL-6 (95% CI 0.51–0.76) and 0.66 for TNFα (95% CI 0.54–0.79) (Fig. 6f–h).

## Discussion

In this study we compared the cytokine and chemokine patterns in two infections in which a cytokine storm is thought to play a role in disease pathogenesis. We found similarities between the cytokines that are elevated in early illness in those who progress to severe illness but also many differences. Although the levels of IL-6 were high during early illness and in the critical phase in those with DHF as reported in many previous studies^29,30^, the levels in those who progressed to develop severe COVID-19 pneumonia and especially those who succumbed to their illness was several folds higher. Therefore, as shown in many studies^31,32^, IL-6 appears to play a significant role in the pathogenesis of severe COVID-19 compared to DHF. It was shown that those with severe pneumonia were significantly more likely to have IL-6 levels of > 7 ng/L (> 7 pg/ml)^31^. Except for 1 patient, all other (7/8) patients had IL-6 levels above this value in severe COVID-19, whereas only one person (1/15) with mild infection had IL-6 levels > 7 ng/L. In contrast, 3/7 patients with DF and 5/7 patients with DHF had IL-6 values exceeding 7 ng/L. However, the levels of IL-6 in those who developed severe COVID-19 were significantly higher than in patients with DHF, and in one of the patients who succumbed to the illness had values of 2179.7 ng/L, 24 h before death. IL-6 acts on many different cell types resulting in inducing production of other inflammatory cytokines such as IL-8, VEGF, MCP-1 and reduced E-cadherin expression, resulting increased endothelial permeability, contributing to ARDS^33^. Although increase vascular permeability resulting in plasma leakage and shock is the main pathological feature of DHF, IL-6 appears to contribute less to disease pathogenesis of DHF compared to COVID-19. However, all these changes in cytokines and chemokines were assessed using a relatively low number of patients and therefore, studies with larger sample sizes are required to further assess these changes.

An impaired type I and type III IFN response in early infection has been shown in COVID-19, especially in older individuals and in those who progress to develop severe disease^4^. We too observed that IFNγ levels were lowest in early illness especially in those who subsequently succumbed to their illness. Although such differences were not observed in those who subsequently developed DHF compared to those with DF, due to the small sample size in this study, it would be important to further characterize these IFN responses in a larger cohort of individuals. However, the striking observation between those who progressed to develop severe COVID-19 vs DHF was the IL-10 levels. It was shown that those who developed severe COVID-19, were significantly more likely to have IL-10 levels of > 9.1 ng/L, which was seen in 35.8% of those with severe disease. Interestingly, while only 2/15 patients with COVID-19 had levels exceeding > 9.1 ng/L, all patients with both DF and DHF had levels far above this value. In fact the serum IL-10 levels in early illness in those who progressed to develop DHF were 25 fold higher than in those who developed severe COVID-19. Serum IL-10, IL-6 and TNFα levels were shown to negatively correlate with the T cell numbers and IL-10 was shown to associate with T cell exhaustion, suggesting a role in disease pathogenesis^34^. We previously showed that serum IL-10 levels inversely correlated with T cell numbers in acute dengue and was associated with T cell apoptosis^11^. In addition, IL-10 was also shown to suppress cytokine production and other antiviral responses by T cells in acute dengue^35^.

As IL-10 was found to be a key cytokine elevated during early illness during acute dengue, we compared the usefulness of IL-10 in predicting those who are likely to develop DHF, along with other key cytokines such as IL-6, TNFα and IL-1β, which cause vascular leak^28,33,36^. Interestingly, only IL-10 was significantly higher in those who proceeded to develop DHF compared to those with DF during early illness. However, the AUC value was not satisfactory (0.73) for it to be used alone as a predictive marker. Although the exact source of IL-10 is not known in acute dengue, given that high levels are present at early illness, it could be produced from monocytes and other immune cells^37,38^. We recently showed that monocytes of healthy individuals who had DHF in the past produced significantly higher viral replication and levels of IL-10, IL-6 and IL-1β compared to those who had mild disease in the past^38^. Given that early appearance of DENV-specific T cell responses are associated with milder clinical disease in acute dengue^39^, production of high levels of IL-10 during early illness, could be one of the key factors leading to severe disease by suppressing antiviral immunity. In fact, dengue NS1 antigen has been shown to cause disease pathogenesis by inducing cytokine production and also by inducing vascular leak^40,41^. NS1 was also shown to induce IL-10 production by monocytes and the persistence of NS1 antigenaemia in patients with acute dengue, correlated with the persistent high levels of IL-10^42^. Collectively these data suggest that while IL-10 is likely to play a significant role in disease pathogenesis in both COVID-19 and dengue, the contribution to pathogenesis of severe dengue may be greater.

Soluble CD40L in serum has been shown to be almost exclusively derived from platelets, which activate CD40 bearing cells to produce cytokines, chemokines and lipid mediators^43^. Increase in sCD40L is associated with an increased risk of thrombotic effects and acute lung injury^43^. Thromboembolic events such as deep vein thrombosis and pulmonary embolism have been frequently reported in patients with severe COVID-19 along with ARDS^44^. Therefore, high levels of sCD40L especially during the critical phase of the illness could be contributing to the occurrence of these complications. On the other hand, sCD40L was significantly higher in patients with DF than DHF during the febrile and the critical phase, possibly as patients with DHF have significantly less platelet counts than patients with DF.

In summary, we assessed the changes in several cytokines and chemokines in patients with varying severity of acute dengue and COVID-19 during different time points in illness. Those who developed severe pneumonia in COVID-19 had high levels of many inflammatory cytokines and chemokines but low IFNγ levels. Patients who proceeded to develop DHF also had high cytokine and chemokine levels, but most strikingly very high IL-10 levels. Low IFNγ response to the SARS-CoV2 and high levels of immunosuppressive cytokines such as IL-10 in both COVID-19 and dengue during early illness is likely to result in an altered antiviral response.

## Methods

### Recruitment of patients

All patients were recruited from National Institute of Infectious Diseases (NIID), Sri Lanka, during acute stage of infection following informed written consent.

#### Patients with COVID-19

Those who had a confirmed SARS-CoV2 infection based on the positive RT-PCR were included in the study following informed written consent. Clinical disease severity was classified as mild, moderate and severe according to the WHO guidance of COVID-19 disease severity^45^. Accordingly, those who had a confirmed symptomatic SARS-CoV2 infection with either fever, cough, fatigue, anorexia, myalgia and shortness of breath with non-specific symptoms such as sore throat, headache and diarrhoea, but with no evidence of hypoxia or pneumonia were classified as having mild illness (n = 15)^45^. Those with clinical signs of pneumonia with a respiratory rate of > 30 breaths/min, or with SpO_2_ < 90% on room air were considered as having severe pneumonia (n = 08)^45^. We did not include any patients with moderate illness as defined by those with clinical and radiological features of pneumonia, but who did not fullfill the criteria of severe pneumonia. Those with mild illness, who had virus shedding for over 35 days and therefore, hospitalized for over 35 days were considered to have prolonged shedding of the virus. A group of those with mild illness, but prolonged shedding of the virus was included in the analysis to determine if early cytokine responses could associate with impaired ability to clear the virus. Blood samples were obtained in the first 9 days and during the second to third week (n = 22). Third blood sample was collected after 30 days (n = 11) from those who had severe disease or prolonged shedding as they were hospitalized for over 40 days.

#### Patients with dengue for Luminex studies

Blood samples were obtained from the patients with DF and those who proceeded to develop DHF at the time of recruitment (day 3 to 4 from onset of illness, time point A) and 2 days later (day 5 or 6 since onset of illness, time point B). None of the patients had any evidence of fluid leakage at the time of recruitment. Those who subsequently developed fluid leakage during the course of illness were classified as having DHF, whereas those who had no evidence of fluid leakage were classified as having DF. The presence of fluid leakage was assessed by the presence of pleural effusions or ascites by ultrasound scans or if the haematocrit increased ≤ 20% from the baseline. However, none of those who did not have ultrasonic evidence of fluid leakage (pleural effusions or ascites) has a haematocrit of ≤ 20% from the baseline, therefore, all patients who were classified as DHF had pleural effusions or ascites. Based on the WHO 2011 dengue disease classification, those who developed fluid leakage along with thrombocytopenia were classified as having DHF.

#### Patients with dengue for evaluation of a biomarker

129 adult patients with an acute dengue infection with a duration of illness of ≤ 4 days, were recruited from the outpatient department (OPD) of the hospital or from the wards. All patients were followed up daily at the OPD or in the ward, and their clinical features and the full blood counts were recorded, until they had completely recovered. Those who were assessed at the OPD, were admitted to hospital if they had any criteria for hospital admission. Based on the 2011 WHO dengue disease classification described above, 48.83% (n = 63) had DHF and 51.16% (n = 66) were classified as having DF.

### RT-PCR for detection of SARS CoV-2

Naso/Oro pharyngeal swabs or sputum samples of suspected SARS- CoV-2 patients were lysed and RNA was extracted using QIAmp Viral RNA Mini Kit (Qiagen, USA, Cat: 52,906). Presence of N gene and ORF1ab gene of SARS-CoV2 was detected with Da An Gene real time PCR kit (Da An Gene, China. Cat: DA-930) by real time RT PCR according to manufacturer’s instructions in ABI 7500 real time PCR system (Applied Biosystems, USA).

### Determining the dengue virus serotype and the viral loads

Viral RNA was extracted using QIAmp Viral RNA Mini Kit (Qiagen, USA, Cat: 52,906) and transcribed into cDNA using a High capacity cDNA reverse transcription kit (Applied Biosystems, USA, cat: 4,368,814). Four gBlock fragments (Integrated DNA Technologies, USA) with known copy numbers were used to generate the standard curve. Oligonucleotide primers, dual labeled probes for DEN 1 to 4 serotypes (Life Technologies, India) and TaqMan Multiplex Master Mix (Applied Biosystems, USA, Cat: 4,461,881) were used for the multiplex quantitative real-time PCR in ABI 7500 real time PCR system (Applied Biosystems, USA).

### Quantification of cytokines and chemokines in patients with dengue and COVID-19

Concentration of seventeen analytes (CD40 Ligand, GM-CSF, IFN-γ, IL-1β, IL-2, IL-4, IL-5, IL-6, IL-10, IL-12p70, IL-13, IL-15, IL-17A, IL-17E, IL-33, MIP-3α AND TNF-α) were assessed in serum samples by Luminex bead-based Th9/Th17/Th 22 discovery performance assay (R&D Systems, USA, Cat:LKTM009) according to manufacturer’s instructions. The plate was read by using the Luminex Magpix analyzer (R&D Systems, USA).

### Quantification of cytokines in patients for biomarker analysis

Serum samples were diluted in sample diluent (SD 13) and loaded into cartridges with relevant high and low control concentrates. Multiplex and single plex ELLA cartridges were run for IL-10, IL-1β, IL-6, TNF-α (Protein Simple, USA, Cat: SPCKA-PS-003043, SPCKB-PS-000216, SPCKB-PS-000276) with their high and low control concentrates IL-10 (Cat:895,003), IL-1β (Cat:894,962), IL-6 (Cat:895,003) and TNF-α (Cat:892,540) in ELLA analyzer (ELLA version 3.4.0.52, Protein Simple, USA) Measurements were taken in triplicates in separate glass nanoreactors (GNRs) for each cytokine of each sample in pg/ml.

### Statistical analysis

Data was analysed by GraphPad Prism 8 version 8.4.2 and R software version 3.3.3 and R Studio version 2.15.2. Four colour coded heatmaps were generated using the 'heatmap2′ function under the 'gplots' package in R software version 3.3.3 and R Studio version 2.15.2. The differences in cytokines in samples were assessed using the two tailed Mann–Whitney U-test and Kruskal–Wallis test. The degree of association between cytokines in dengue patients with viral loads was analyzed using Spearman correlation coefficient test. A receiver-operator characteristic (ROC) curves showing the area under the curve (AUC) were generated to determine the discriminatory performance of IL-10 and other cytokines in predicting those who will develop DHF during early illness.

### Ethical approval

Ethical approval was received by the Ethics Review Committee of Faculty of Medical Sciences, University of Sri Jayewardenepura. The study on humans was carried out in accordance with relevant guidelines and regulations (The Declaration of Helsinki).

## Supplementary information

Supplementary Information.

## Data Availability

Data is available within the manuscript and the supplementary files.
