# Supplementary material for: Similarities and differences between the ‘cytokine storms’ in acute dengue and COVID-19
Source: Sci Rep. 2020 Nov 16;10:19839. doi: 10.1038/s41598-020-76836-2 (PMC7670444; doi:10.1038/s41598-020-76836-2)
Supplement: Supplementary file 1 — Supplementary Information. [file 41598_2020_76836_MOESM1_ESM.pdf]

Supplementary data

Similarities and differences between the 'cytokine storms' in acute dengue and COVID-19

Shashika Dayarathna[1#], Chandima Jeewandara[1,3#], Laksiri Gomes[1], Gayasha Somathilaka[3], Deshni Jayathilaka[1], Vimalahan Vimalachandran[1], Ananda Wijewickrama[2], Eranga Narangoda[2], Damayanthi Idampitiya[2], Graham S. Ogg [1,4], Gathsaurie Neelika Malavige[1,4*]

Centre for Dengue Research, Faculty of Medical Sciences, University of Sri Jayawardenapura, Sri Lanka[1]; National Institute of Infectious Diseases, Angoda, Sri Lanka[2]; Allergy, Immunology and Cell Biology Unit, University of Sri Jayewardenepura[3]; MRC Human Immunology Unit, MRC Weatherall Institute of Molecular Medicine, University of Oxford, Oxford, United Kingdom[4]

Supplementary table 1: Cytokine and chemokine levels in patients with different clinical disease severity at different time point in infection

Cytokine	Time point in illness	COVID-19 Severe pneumonia N= 8	COVID-19 Mild illness N=15	DF N=7	DHF N=7
IFNγ	A<br>B	3.25 (1.7 to 13.52)<br>8.81 (2.74 to 14.45)	11.56 (7.8 to 12.83)<br>10.3 (6.66 to 15.43)	20.10 (12.58 to 20.78)<br>11.56 (3.49 to 20.1)	17.45 (9.99 to 20.78)<br>18.08 (15.42 to 18.08)
IL-10	A<br>B	34.9 (29 to 65.46)<br>44.06 (23.11 to 96.25)	5.98 (3.31 to 11.57)<br>5.98 (5.98 to 7.36)	115.05 (34.97 to 287.49)<br>522.65 (217.88 to 762.98)	907.65 (682.29 to 1786.05)<br>405.19 (128.58 to 866.56)
IL-6	A<br>B	22.65 (8.46 to 113.02)<br>207.34 (20.94 to 574.63)	2.06 (0.81 to 8.89)<br>1.04 (0.58 to 1.54)	2.06 (2.06 to 4.64)<br>3.16 (2.61 to 10.83)	11.37 (5.40 to 84.17)<br>18.56 (7.63 to 72.84)
MIP3α	A<br>B	5.89 (4.03 to 11.72)<br>9.32 (6.4 to 14.7)	6.91 (2.7 to 12.24)<br>4.54 (1.87 to 5.46)	4.20 (3.20 to 6.40)<br>4.20 (2.70 to 5.04)	11.47 (9.78 to 30.41)<br>6.57 (6.23 to 8.28)
CD40L	A<br>B	3638.11 (2922.41 to5067.21)<br>7507.53 (5847.71 to 8560.72)	3720.62 (2740.9 to 4721)<br>3886.05 (2218.96 to 4427.24)	3064.52 (2600.66 to 3289.12)<br>2139.46 (1804.11 to 3270.06)	1551.04 (480.07 to 1854.34)<br>1166.06 (1014.54 to 1318.93)
TNFα	A<br>B	8.48 (1.65 to 10.82)<br>4.06 (2.41 to 8.03)	0.89 (0.22 to 3.66)<br>0.89 (0.22 to 1.63)	6.67 (5.79 to 7.57)<br>7.57 (4.54 to 8.95)	14.18 (9.65 to 73.89)<br>12.23 (7.20 to 110.97)

IL-13	A<br>B	23.9 (21.01 to 23.9)<br>23.9 (21.01 to 24.59)	23.9 (21.01 to 23.9)<br>23.9 (23.9 to 25.28)	23.90 (22.45 to 23.90)<br>23.90 (21.01 to 23.90)	22.45 (21.01 to 23.90)<br>21.01 (19.48 to 21.01)
IL-15	A<br>B	1.13 (0.82 to 1.76)<br>1.44 (0.54 to 1.92)	0.54 (0.35 to 0.92)<br>0.54 (0.22 to 0.73)	1.76 (1.54 to 2.31)<br>1.13 (0.63 to 1.33)	2.54 (1.28 to 4.67)<br>1.13 (0.83 to 1.60)
IL-1β	A<br>B	3.14 (1.89 to 4.39)<br>1.63 (0.64 to 30.75)	0.16 (0.16 to 0.16)<br>* All the values were undetectable	* All the values were undetectable<br>0.16 (0.16 to 0.16)	* All the values were undetectable<br>2.64 (1.64 to 3.63)
IL-2	A<br>B	1.06 (0.61 to 3.54)<br>3.08 (1.2 to 14.97)	0.8 (0.31 to 1.93)<br>0.8 (0.31 to 1.88)	0.31 (0.31 to 0.67)<br>0.55 (0.31 to 1.61)	1.33 (0.80 to 1.61)<br>0.31 (0.31 to 0.80)
IL-5	A<br>B	1.45 (1.12 to 19.35)<br>1.62 (1.12 to 2.11)	1.12 (1.12 to 1.45)<br>1.45 (1.45 to 1.45)	1.12 (1.12 to 1.45)<br>1.12 (1.12 to 1.45)	1.12 (1.12 to 1.33)<br>1.12 (1.12 to 1.12)
IL-33	A<br>B	6.6 (3.49 to 9.71)<br>1.00 (0.39 to 1.94)	0.39 (0.39 to 0.39)<br>1.45 (1.45 to 1.45)	1.00 (0.69 to 1.31)<br>1.62 (1.00 to 2.27)	4.27 (1.62 to 12.82)<br>1.65 (1.02 to 2.29)

Supplementary Table 2. Clinical and Laboratory features of dengue patients recruited for identification of cytokine biomarkers

Characteristic	DF admitted (n=36)	DF Not Admitted (n=30)	DHF (n=63)
Fever	36 (100%)	29 (96.67%)	61 (96.83%)
Abdominal pain	3 (8.33%)	5 (16.67%)	21 (33.33%)
Vomiting	3 (8.33%)	4 (13.33%)	15 (23.81%)
Bleeding manifestations	2 (5.56%)	0	11 (17.46%)
Pleural effusions	0	0	8 (12.70%)
Ascites in Hepatorenal pouch	0	0	52 (82.54%)
Hepatomegaly	0	0	3 (4.76%)
Platelets <20,000	0	0	33 (52.38%)
20,000-50,000	9 (25.00%)	1 (3.33%)	28 (44.44%)
50,000-100,000	13 (36.11%)	1 (3.33%)	2 (3.17%)
>100,000	14 (38.89%)	26 (86.67%)	0
Lymphocytes <750	19 (52.78%)	8 (26.67%)	30 (47.62%)
750-1500	14 (38.89%)	10 (33.33%)	21 (33.33%)
>1500	3 (8.33%)	8 (26.67%)	12 (19.05%)
DEN 1	10 (27.78%)	16 (53.33%)	8 (12.70%)
DEN 2	18 (50.00%)	13 (43.33%)	41 (65.08%)
DEN 3	7 (19.44%)	1 (3.33%)	9 (14.29%)
Dual infection	1 (2.78%)		1 (1.59%)
